# The “Forgotten Grievers”: The Impact of Pupil Suicide on Post-Trauma and Grief Symptoms in School Staff

**DOI:** 10.3390/ijerph191912160

**Published:** 2022-09-26

**Authors:** Noa Tiech Fire, Yari Gvion, Sarit Alkalay, Gil Zalsman

**Affiliations:** 1 Ministry of Education, Jerusalem 9510557, Israel; 2 Department of Psychology, Bar Ilan University, Ramat Gan 5290002, Israel; 3 Department of Psychology, Yezreel Valley Academic College, Yezreel Valley 1930600, Israel; 4 Geha Mental Health Center, Petah Tikwa 49100, Israel; 5 Sackler Faculty of Medicine, Tel Aviv University, Tel Aviv 6997801, Israel; 6 Division of Molecular Imaging and Neuropathology, Department of Psychiatry, New York State Psychiatric Institute, Columbia University, New York, NY 10032, USA

**Keywords:** suicide, school staff, grief, trauma

## Abstract

**Highlights:**

- Following a suicide of a pupil principals and home-room teachers suffer more from complicated grief and PTSD compared to psychologists and counsellors.
- Principals and home-room teachers should receive more preparatory training.

**Abstract:**

Background: The suicide of a pupil impacts survivors greatly, but most studies on the subject do not consider school staff, and do not differentiate between the various professional domains. Our aim was to investigate the existence of differences in symptoms of complicated grief as well as post-trauma symptoms after a pupil’s suicide, among school staff in four domains: counsellors, psychologists, principals and home-room teachers. Method: Eighty-four staff members from schools that lost pupils to suicide within the past five years were assessed for symptoms of complicated grief and trauma. All reported their symptoms using self-report scales. Results: Principals and home-room teachers had significantly higher complicated grief and post-trauma symptoms. The main limitations of this study are that the data were collected via self-report questionnaires, which can introduce bias. Additionally, the sample is relatively small and comprises mainly women. Conclusions: School professionals in domains receiving less coping and crisis training, and those with supervisory responsibilities (principals and home-room teachers) show more symptoms of trauma and complicated grief after a pupil’s suicide, and require special attention. More preparatory training would surely benefit them and assist them in coping with such crises.

## 1. Introduction

A pupil’s suicide creates extensive circles of vulnerability, not only among the pupil’s family members, but also among their classmates, guides, teachers and other acquaintances [1]. Following the death of a pupil, school staff are at the frontline, having to manage the acute crisis. Still, they are often forgotten and not considered among those impacted by the death and needing support [2]. Only in recent years has it been recognized that this subject must be investigated and talked about in order to prevent more such occurrences [3]. 

Every suicide leaves behind approximately 25 people who were close to the person who died by suicide and are at greater risk of developing psychopathologies after the event [1,4,5], including complicated grief, depression, suicide and post-trauma symptoms [6]. Most studies so far have investigated families and friends of the person who died by suicide. Other studies investigated psychiatrists and therapists. Among other things they found that women reported a greater effect on their clinical confidence [7], and that younger, less-experienced clinicians were more affected by a patient’s suicide than older clinicians with more experience [8]. Many studies emphasize the need for social and professional support, which includes opportunities to talk about the suicide experience with others—especially with senior professionals and with those who have also experienced suicide in their family or work. This support is common among mental health practitioners [9]. However, little attention has been paid in the literature to the impact of a pupil’s suicide on school staff, and to their need for support and training to cope with a pupil’s suicide [1]. 

It seems reasonable that staff members who have been trained for coping with crises would react differently from staff members who have had no such training. Indeed, it was found that staff members who were trained to cope with emergencies, stress and crises reported less emotional overload, avoidance and detachment in times of school crises than un-trained staff [10]. Moreover, trained personnel demonstrated more competence and reported more satisfaction and a sense of emotional growth after offering support to other grievers [10]. 

The aim of the current study was to investigate the existence of differences in complicated grief and post-trauma symptoms after a pupil’s suicide, among school staff in four domains: counsellors, psychologists, principals and home-room teachers. Given the nature of their professions, school counsellors and psychologists undergo training to cope with crises and loss—their patients’ as well as their own. They are also trained and prepared to cope with the impact a patient’s crisis has on themselves. Meanwhile, principals and home-room teachers often do not receive such training, and even when they do, it is not as thorough [11]. We hypothesized that psychologists and counsellors will report less symptoms of complicated grief and PTSD compared to principles and home teachers. The research hypotheses were tested in relation to background variables of gender and seniority.

To the best of our knowledge, this is the first study to examine these four domains in the context of pupil suicide.

## 2. Materials and Methods

### 2.1. Subjects 

We located all the schools in Israel at which a pupil had died by suicide during the previous five years (*n* = 29). All schools were middle schools or high schools. At each school the school counsellor, the school psychologist, the principal and the home-room teacher of the pupil who died by suicide were prospective participants (*n* = 116). In this way we identified the entire population of potential participants and attempted to speak with all of them. Six potential participants (5.2%) refused to participate, claiming that the subject was too emotionally difficult. Twenty-six potential participants (22.4%) could not be located. They no longer worked at the school where the incident occurred, and were not found in the Ministry of Education database, implying that after the incident they had left the profession altogether. Eighty-four (72.4%) agreed to participate, of whom sixty-one (73%) were women. This gender distribution is similar to the general distribution of educational staff in Israel (80% females) [12]. Within the four domains, out of 14 principals, 8 were men; out of 17 home-room teachers, 4 were men, out of 27 counsellors, 3 were men; and out of 26 psychologists, 8 were men. The participants’ average age was 47.3 (S.D. 8.8), and their average seniority was 18.8 years (S.D. 8.6). These demographic characteristics are compatible with data for Israeli school staff: sixty percent are aged 30–49, and sixty percent have more than ten years of seniority [12]. With regard to the different professions, we interviewed school counsellors (*n* = 27, 32%), school psychologists (*n* = 26, 31%), principals (*n* = 14, 17%) and home-room teachers (*n* = 17, 20%), all having encountered cases of pupils who died from suicide. 

### 2.2. Procedure

Upon receiving approval from the Israel Ministry of Education’s institutional review board, we searched the Ministry of Education’s database for schools at which a student had died by suicide during the previous five years (*n* = 29). After participants signed an informed consent form, they received a link to a set of computerized rating scales. Participants were asked to answer the questionnaires regarding the student’s suicide event. Scales were administered in Hebrew. Researchers were blind to the individual rating scale responders.

### 2.3. Tools

**Post-traumatic Diagnostic Scale (PDS)** [13]—This is a widely used self-report scale for symptoms of post-traumatic stress disorder (PTSD) based on the DSM IV. The scale includes 49 items (Cronbach α = 0.92). 

**Inventory of Traumatic Grief (ITG)** [14]—The short self-report version that includes 14 items (Cronbach α = 0.95) was used in this study. It covers each of the symptoms of traumatic grief as well as other potentially maladaptive symptoms of grief.

### 2.4. Statistical Methods

SPSS software (IBM Corp. Version 23.0) was used for statistical analyses. As for the normal distribution of the measures, inter-group differences were analyzed using ANOVA. Post hoc Bonferroni analysis was used to control for multiple testing. The significance level was set at 0.01, and all analyses were two-tailed.

Note that our research design is suitable for a multi-level analysis, whereby one variable is nested within another broader variable (group-level variable) [15]. Specifically, all participants from a particular school were considered part of the same group and might have been affected by the same context variable. However, we could not use multilevel model (HLM) analysis to explore this nesting effect for two reasons. The first relates to the anonymity requirement of the ethics board. We could not match the participants to a specific school (see the ethical consideration section). The second reason relates to the small number of participants from each school. Our research design imposed that a maximum of four potential participants from each school would be approached—the home-room teacher, the school principal, the school counsellor and the school psychologist. It is suggested that “the most commonly offered rule of thumb with regard to sample size for multilevel models is at least 20 groups and at least 30 observations per group” [16] (p. 272). Thus, although 29 schools were located, which satisfies the requirement for the number of groups, each school (i.e., group) consisted of less than 30 observations as required. 

## 3. Results

**Figure 1:**
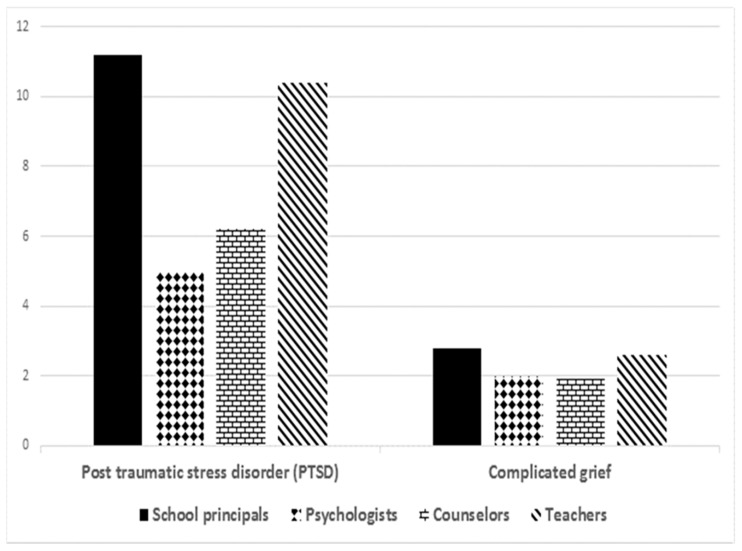
Differences between the domains regarding the intensity of complicated grief and post-traumatic stress disorder (PTSD). The scales of the measures were adjusted so that it would be possible to put them on the same graph in relation to each other. *p* < 0.01.

Following a pupil’s suicide, significant differences in symptoms of PTSD (F_(3,83__)_ = 5.90, *p* = 0.001) and complicated grief (F_(3,79)_ = 14.53, *p* = 0.001) were demonstrated across the four school staff domains. Both complicated grief and PTSD measurements of school principals (M = 2.69, SD = 1.11; M = 11.14, SD = 10.61) and home-room teachers (M = 2.65, SD = 1.03; M = 10.32, SD = 9.02) were found to be significantly higher than those of counsellors (M = 1.89, SD = 0.60; M = 6.26, SD = 8.54) and psychologists (M = 1.94, SD = 0.63; M = 5.16, SD = 7.5) (Figure 1). No differences were found between the groups regarding gender or seniority, (*p* > 0.01), but more PTSD symptoms were found among males than among females (t_(2,54)_ = 3.08, *p* = 0.002). Male participants reported higher symptoms of PTSD compared to females (M = 2.11, SD = 1.55 vs. M = 1.50, SD = 1.24). Unfortunately, due to the small sample size we could not test the interaction of profession and gender with regard to PTSD.

## 4. Discussion

This study demonstrated significant differences in symptoms of PTSD and complicated grief among four domains of school staff: principals, counsellors, psychologists and home-room teachers. Principals and home-room teachers reported higher levels of complicated grief and PTSD symptoms than psychologists and counsellors. 

Previous studies found a pupil’s death to negatively influence the mood of other students and of school staff [17,18]. However, these studies did not focus specifically on suicide, while studies that investigated pupils’ suicides did not focus on the specific staff domains discussed in the current study. To the best of our knowledge, this is the first study to assess the coping of these four school staff domains and their emotional parameters after a pupil’s suicide. 

The literature considers school staff to be “forgotten grievers” [19] since they feel deep loneliness after the loss of a student but are not treated as parties impacted by the death. According to the studies, these people are at high risk for PTSD and complicated grief [6,19]. 

The findings of the current study support these reports and add a hierarchical structure of symptoms on which the four school staff domains can be placed. Principals and home-room teachers focus on pedagogical and educational skills as well as procedures and organization [20]. They are less involved in situations that require emotional support, and receive less training on the subject. Therefore, they are more likely to be in greater distress when dealing with an event such as the suicide of a pupil.

The confidence of home-room teachers in assisting students with mental health problems is associated with the home-room teachers’ subjective psychological well-being, their knowledge and capability to comprehend children’s mental health issues and with their satisfaction with the overall school climate [21]. In times of crisis, they are concerned with organization strategy, control and returning to routine [11]. Since they do not feel qualified to treat mental health problems, and because they are engaged in organization strategy and helping pupils return to routine, when a suicidal event occurs in their classroom, their emotional response to the situation is more severe. For example, in the current study, a theme that arose on the part of the subjects was the lack of support from the education system and the feeling that they had been “abandoned” to such a difficult situation; one educator stated: “No support was given to the counselor or the teaching staff who knew the student”. “We were left to cope alone”. A school principal reported: “The ministry (of education) must provide a solution for educators and caregivers who have experienced the loss of a student, this is a work accident in every respect. On the day of the event, I felt wrapped and so did in the first week. After that, I was left in great solitude and with the feeling that everyone expects us to return to normal as if it never happened. However, this was not the case”. A counselor reported: “Unfortunately, I did not feel emotional support for my work as a counselor and no additional professional help was provided for the incident. The feeling is ‘alone in the battle’”.

Another possible explanation for our findings is that, during a crisis such as the death of a pupil, home-room teachers tend to be closer to the students and to the family and friends of the pupil who died by suicide compared to other staff members [22]. The degree of closeness to the person who died by suicide has been shown to have an impact on PTSD symptoms [23]; thus, the greater the proximity to the students, the higher the symptoms of the trauma.

According to the rules of the Israeli Ministry of Education, psychologists and counsellors are responsible for the well-being and mental health of pupils [24]. They are trained to cope with crises and distress, and are also under ongoing professional supervision throughout the school year. 

Interestingly, male participants of our study reported higher levels of PTSD compared to female participants. Due to the small sample of men we could not test the interaction of gender and profession. A plausible explanation for our findings is that, in a country where military service is compulsory and men are commonly assigned to combat roles, men more than women are exposed to traumatic events. Research shows that repeated trauma impedes teachers’ and counselors’ adaptive coping following a student’s suicide [5]. This is in line with findings from another of our projects [25] suggesting that school staff members who previously experienced the suicide or attempted suicide of someone close should be considered a risk group for lower levels of coping strategies.

## 5. Limitations

The retrospective approach of this study, evaluating the impact of past events and the subjective nature of the measurements used (self-reporting) may introduce recall biases. In addition, the relatively small size of the study population did not allow consideration of the impact of time elapsed since the suicide. There is no doubt that the current impact of a recent loss differs significantly from the current impact of a loss that occurred years earlier. Other limitations concern the study’s sample. Our sample consists mostly of women (73%), reflecting the fact that most school employees in Israel are women [12]. Thus, further research should be conducted with a larger sample of men to examine the replicability of our results and their generalizability to the entire population. The small number of men did not allow us to test for interaction with regard to PTSD; it is recommended that this interaction is tested in future studies. 

Additionally, 27% of the potential participants did not participate. Most of these potential participants who did not participate could not be located because they no longer worked at the school at which the incident occurred, and we were unable locate them in other ways. Some potential participants refused to participate, claiming the topic was too painful for them. The low rate of this explicit refusal (5%) is lower than in other social science studies [26]. Yet, those who refused to participate or who were not located may have exhibited a different pattern of responses than the pattern exhibited by our participants, and their absence may have influenced the outcomes of our study. Lastly, our study did not examine additional background variables that have the potential to interfere, such as a previous psychopathological situation. Nor did we measure the duration of contact or emotional proximity of study participants to the pupils who died from suicide. However, it is worth mentioning that in the Israeli education system, the home-room teacher is the staff member the student meets most often. The counselor, psychologist and principle are usually background figures. 

## 6. Conclusions 

The policy of the Stress Unit in the Ministry of Education emphasizes the need to provide intensive counseling and accompaniment for staff. However, its focus is on the staff being able to provide these responses to the students, and there is no accompaniment and support for the staff themselves. Decision makers must give special consideration to the severe impact of a pupil’s suicide on principals and home-room teachers, who are more vulnerable to mental health symptomatology following such an occurrence. Improving coping skills and establishing prevention and support programs for school staff may assist them in better coping with these difficult situations. Thus, similar to psychologists and counselors who undergo training to cope with crises and loss, it is suggested that home-room teachers and principals receive training focused on coping with the crisis of a pupil’s death by suicide and the impact this crisis has on themselves.

## Data Availability

Upon request.

## References

[1] Levi-Belz Y. (2015). Stress-related growth among suicide survivors: The role of interpersonal and cognitive factors. Arch. Suicide Res..

[2] Swihart J., Silliman B., McNeil J. (1992). Death of a student: Implications for secondary school counselors. Sch. Couns..

[3] Wasserman D., Hoven C.W., Wasserman C., Wall M., Eisenberg R., Hadlaczky G., Kelleher I., Sarchiapone M., Apter A., Balazs J. (2015). School-based suicide prevention programmes: The SEYLE cluster-randomised, controlled trial. Lancet.

[4] McLoughlin A.B., Gould M.S., Malone K.M. (2015). Global trends in teenage suicide: 2003–2014. QJM.

[5] Cerel J., Maple M., van de Venne J., Moore M., Flaherty C., Brown M. (2016). Exposure to Suicide in the Community: Prevalence and Correlates in One, U.S. State. Public Health Rep..

[6] Cerel J., Padgett J.H., Conwell Y., Reed G.A. (2009). A call for research: The need to better understand the impact of support groups for suicide survivors. Suicide Life Threat. Behav..

[7] Gibbons R., Brand A., Carbonnier A., Croft A., Lascelles K., Wolfart G., Hawton K. (2019). Effects of patient suicide on psychiatrists: Survey of experiences and support required. BJPsych Bull..

[8] Chemtob C.M., Hamada R.S., Bauer G., Kinney B., Torigoe R.Y. (1988). Patients’ suicides: Frequency and impact on psychiatrists. Am. J. Psychiatry.

[9] Sandford D.M., Kirtley O.J., Thaites R., O’Connor R.C. (2021). The impact on mental health practitioners of the death of apatient by suicide: A systematic review. Clin. Psychol. Psychother..

[10] Christianson C.L., Everall R.D. (2009). Constructing bridges of support: School counsellors’ experiences of student suicide. Can. J. Coun. Psychother..

[11] Avney Rosha Institute Expected Outcomes from School Principals at the Start of Their Career. http://www.avneyrosha.org.il/eng/Documents/Expected_outcomes.pdf.2010.

[12] Zarad E. (2019). Knesset Report on Teachers in the Israeli Educational System. https://fs.knesset.gov.il/globaldocs/MMM/fa83866a-6e5b-e911-80e9-00155d0aeebb/2_fa83866a-6e5b-e911-80e9-00155d0aeebb_11_13503.pdf.

[13] Foa E.B. (1996). Posttraumatic Diagnostic Scale Manual.

[14] Prigerson H.G., Bierhals A.J., Kasl S.V., Reynolds C.F., Shear M.K., Newsom J.T., Jacobs S. (1996). Complicated grief as a disorder distinct from bereavement-related depression and anxiety: A replication study. Am. J. Psychiatry.

[15] Cooksey R.W. (2020). Illustrating Statistical Procedures: Finding Meaning in Quantitative Data.

[16] Bickel R. (2007). Multilevel Analysis for Applied Research.

[17] Lazenby R.B. (2006). Teachers Dealing with the Death of Students: A Qualitative Analysis. J. Hosp. Palliat. Nurs..

[18] Munson L.J., Hunt N. (2005). Teachers grieve! What can we do for our colleagues and ourselves when a student dies?. Teach. Except. Child..

[19] Rowling L. (1995). The disenfranchised grief of teachers. OMEGA-J. Death Dying.

[20] Oplatka I. (2009). Learning the principal’s future internal career experiences: An assessment of a unique principal preparation programme in Israel. Int J. Educ. Manag..

[21] Sisask M., Värnik P., Värnik A., Apter A., Balazs J., Balint M., Bobes J., Brunner R., Corcoran P., Cozman D. (2013). Teacher satisfaction with school and psychological well-being affects their readiness to help children with mental health problems. Health Educ. J..

[22] Liora F. (2008). School Management Under Conditions of Terror and Ongoing Trauma.

[23] Bonanno G.A., Kaltman S. (2001). The varieties of grief experience. Clin. Psychol. Rev..

[24] Israel Ministry of Education Director General Directive, Educational Counselor Outline of Educational Psychology Services (Hebrew). https://cms.education.gov.il/EducationCMS/Units/Sherut/Takanon/Perek5/Yoetz/.

[25] Tiech-Fire N., Alkalay S., Gvion Y., Zalsman G. (2022). The association between school staff’s coping strategies following a student’s suicide, school climate, and previous experience with suicide. Br. Educ. Psychol..

[26] Anastasi A. (1990). Psychological Testing.

